# Predicting the Response to Non-invasive Brain Stimulation in Stroke

**DOI:** 10.3389/fneur.2019.00302

**Published:** 2019-04-02

**Authors:** Smadar Ovadia-Caro, Ahmed A. Khalil, Bernhard Sehm, Arno Villringer, Vadim V. Nikulin, Maria Nazarova

**Affiliations:** ^1^Department of Neurology, Max Planck Institute for Human Cognitive and Brain Sciences, Leipzig, Germany; ^2^Berlin School of Mind and Brain, Humboldt-Universität zu Berlin, Berlin, Germany; ^3^Neurophysics Group, Department of Neurology, Campus Benjamin Franklin, Charité-Universitätsmedizin Berlin, Berlin, Germany; ^4^Center for Stroke Research Berlin, Charité-Universitätsmedizin Berlin, Berlin, Germany; ^5^Department of Cognitive Neurology, University Hospital Leipzig and Faculty of Medicine, University of Leipzig, Leipzig, Germany; ^6^Bernstein Center for Computational Neuroscience, Berlin, Germany; ^7^Center for Cognition and Decision Making, Institute for Cognitive Neuroscience, National Research University Higher School of Economics, Moscow, Russia; ^8^Federal Center for Cerebrovascular Pathology and Stroke, The Ministry of Healthcare of the Russian Federation, Federal State Budget Institution, Moscow, Russia

**Keywords:** NIBS, stroke, variability, functional connectivity, ongoing neuronal oscillations, long-range temporal correlations, fMRI, EEG

Neuromodulatory non-invasive brain stimulation (NIBS) techniques are experimental therapies for improving motor function after stroke. The aim of neuromodulation is to enhance adaptive or suppress maladaptive processes of post-stroke reorganization.

However, results on the effectiveness of these methods, which include transcranial magnetic stimulation (TMS) and transcranial direct current stimulation (tDCS), are mixed. The results of recent large clinical trials and meta-analyses range from no improvement in motor function (1, 2) to moderate improvement (1–6) at the group level. Though evidence supporting efficacy is better for TMS (7) than for tDCS (6), individual stroke patients' response to NIBS is nevertheless extremely variable (8–11). This is reminiscent of the development of other stroke therapies, such as thrombolysis and mechanical thrombectomy, where early studies were largely mixed before patient selection was refined (12, 13). NIBS in stroke faces a similar challenge of refining patient selection and individualizing protocols to determine its therapeutic potential.

The variable response to NIBS in stroke patients is a byproduct of multiple factors that influence response to NIBS in healthy controls (14, 15), as well as factors that influence the response specifically in stroke patients (8). The former include factors such as age, gender, anatomical variability, intake of stimulant substances, and baseline neurophysiological state but also technical factors such as stimulation intensity, TMS coil orientation, and stimulation duration (16–18). Specifically in stroke patients, symptom severity, size and location of lesions, stroke etiology, and time from symptom onset to intervention influence the response to NIBS as well. Importantly, these different variability-causing factors interact to affect the response to NIBS, such as the potential amplification of inter-individual differences in brain anatomy (19, 20) by stroke lesions (21, 22). Such interactions make understanding the causes of NIBS response variability in stroke challenging.

Although the need for individualized stimulation protocols in stroke patients is widely accepted, it is still unclear exactly how this will be achieved. At the very least, the factors influencing variability in healthy subjects should be controlled as much as possible through appropriate and careful study design (23) and checklist-based reporting of factors during data collection (24). To address the specific factors for stroke, patient selection for NIBS should be informed by pathophysiological processes. This requires that we know which processes are relevant, that we are capable of measuring them, and that we know the optimum timing and patient-related characteristics for treatment administration.

## Models of Reorganization as a Basis for Stimulation Protocols

Until recently, NIBS protocols have mostly been based on the interhemispheric competition model (25, 26), which postulates that the unaffected hemisphere overly inhibits the affected hemisphere. Despite NIBS strategies based on this model being largely ineffective at the group level (27–30), it is still a popular approach used by several recent (9) and ongoing clinical trials. In severely affected patients in particular, the validity of this model has been questioned (31, 32) and an alternative, the vicariation model, suggested (33). The vicariation model postulates that the function of the unaffected hemisphere compensates for the impairment of the affected hemisphere, thereby presenting an adaptive, rather than maladaptive, process (32, 34–37).

These contradictory models have been unified in the bimodal-balance recovery model, taking us a step further to individualized therapy (25). This uses a metric, the “structural reserve,” defined as the integrity of the white matter motor pathways, to determine whether the inter-hemispheric competition or vicariation model is applicable in a given patient. According to the model, in patients with high structural reserve, the over-activation of the unaffected hemisphere is maladaptive, while in patients with low structural reserve, this over-activation is compensatory. Supporting this model, severely affected patients, with presumably low structural reserve, have poorer outcomes when inhibitory NIBS protocols are applied to their unaffected hemispheres (28, 37), emphasizing the need to modify “one-size-fit-all” NIBS protocols.

However, it is yet to be resolved which clinical and imaging characteristics are appropriate proxies for structural reserve. Most evidence thus far comes from studies investigating the ability of these characteristics to predict stroke outcome. White matter integrity, quantified with the fractional anisotropy of white matter tracts on diffusion tensor imaging, is commonly used (38–42). However, a good predictor of stroke outcome (prognostic biomarker) is not necessarily useful for predicting the response to specific NIBS paradigms (selection biomarker) (43). Prognostic biomarkers may provide a good starting point; however, they need to be validated to demonstrate their specific role and relative importance in influencing the response to NIBS after stroke. Two recent promising studies show that behavioral measures such as the Action Research Arm Test and the Fugl-Meyer score are predictors of the response to NIBS in correlation with white matter integrity measured using imaging (44, 45). These studies show that both clinical and imaging measures associated with structural reserve influence the effectiveness of facilitation of the affected hemisphere or inhibition of the unaffected hemisphere, providing direct support for the bimodal-balance recovery model, and setting the ground for future studies validating these selection biomarkers.

On the methodological level, to develop a framework to guide individualized NIBS therapy, large studies with many patients and variables must be conducted (46). The analysis of such large-volume, complex data would be suited for machine learning approaches. Considering preliminary evidence on the high correlation between clinical and imaging-based biomarkers (44, 45), as well as the high correlation within the different clinical features of stroke (47, 48), potential models guiding NIBS therapy need not to be overly complex, and it is likely that highly correlated measures can be reduced to factors of lower dimension that explain substantial variability.

**Figure 1:**
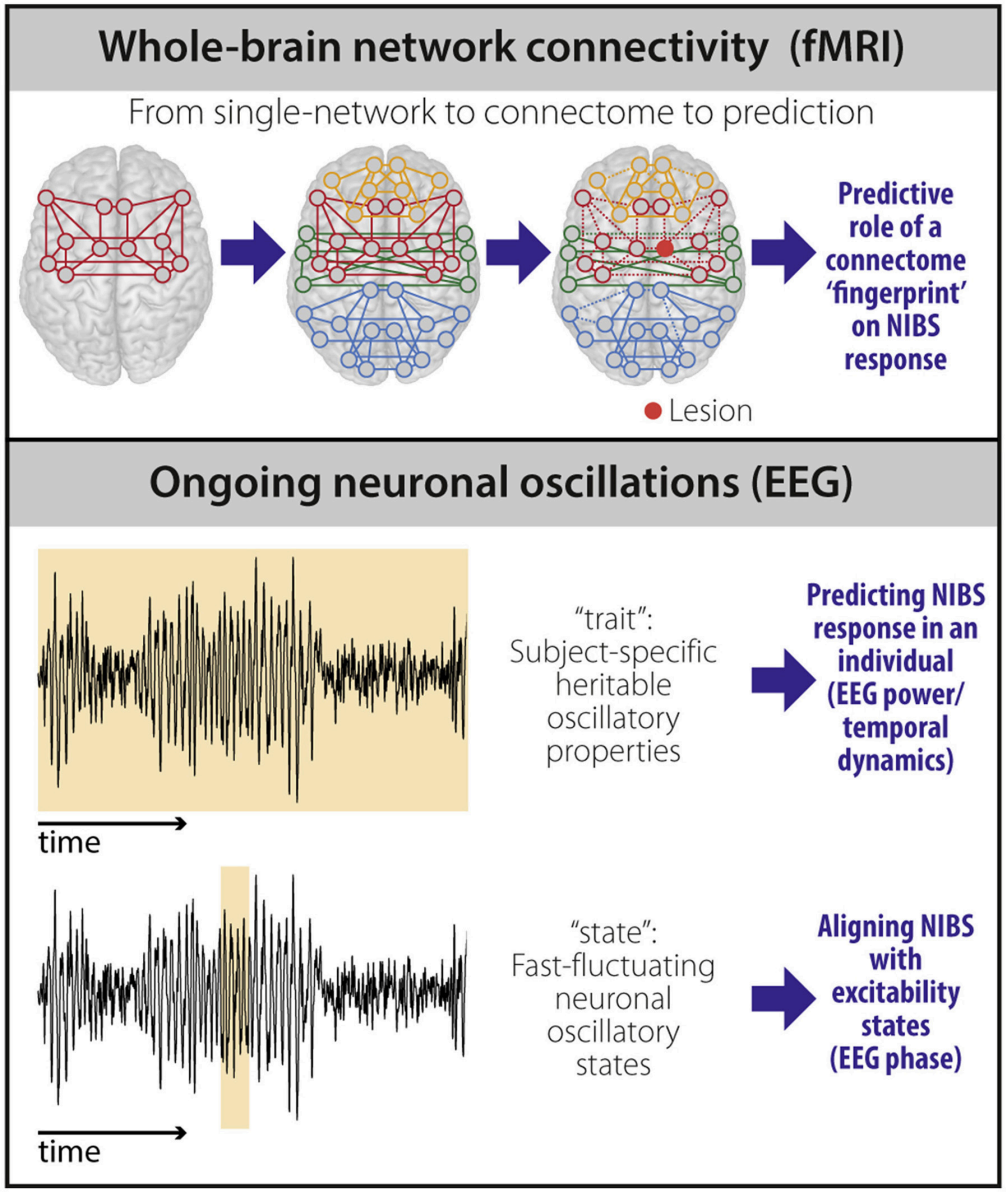
Potential biomarkers to predict NIBS response. fMRI-based connectivity techniques (top) provide information on the brain's large-scale functional organization. Moving beyond the description of single networks, whole-brain (“connectome”) connectivity models capture the heterogeneity and individual reorganization after stroke using a single scan. The individual connectome “fingerprint” could therefore be used as a predictor of NIBS response based on stroke pathophysiology in an individual patient. Properties of ongoing neuronal oscillations measured using EEG (bottom) carry both stable, heritable (“trait”), and transiently changing (“state”) information. EEG power and temporal dynamics can be used as “trait” measures and provide prediction of NIBS response at the individual level. EEG phase can be used to temporally align NIBS stimulation with excitability states to improve NIBS efficacy at the individual level.

Two potential imaging-based biomarkers of NIBS response in stroke—whole-brain connectivity and the brain's propensity to respond to stimulation—have been largely ignored and are addressed here (Figure 1).

## Whole-Brain Network Connectivity

Stroke is not a mere localized phenomenon. Widespread effects of stroke are found within the affected network (49), but also beyond it (50–54), and connectivity has been suggested as the underlying mechanism mediating these indirect effects (33, 55).

Whole-brain connectivity models based on resting-state functional magnetic resonance imaging (rs-fMRI) show that modulation of long-range connections between different regions outside lesions and their changes over time relate to stroke recovery on the individual level (56–58). In addition, most strokes affect multiple behavioral domains and thus changes in multiple functional networks better characterize a single patient. These factors likely contribute to the observed response variability to NIBS, but have not been sufficiently considered thus far, as both connectivity alterations in stroke and NIBS protocols have mostly been investigated in the context of isolated networks (8, 59–62). Given the effects of NIBS on distributed networks (63–65) and the understanding of stroke as a distributed pathology (55, 66, 67), when applying stimulation in these patients, assuming that a single functional network is being, or indeed should be, targeted is problematic.

Whole-brain connectivity using rs-fMRI is well-suited for use in patients because it captures, with a single task-free scan, information on functional connectivity of multiple brain networks (55, 66). In our opinion, this approach can be used to develop more realistic models of spontaneous reorganization after stroke, and could prove beneficial for designing individualized stimulation protocols.

A methodological limitation of connectivity approaches is that they rely on a-priori delineation of somewhat arbitrary boundaries between networks. Dimensionality reduction of whole-brain connections overcomes this problem (68). Using this data-driven approach, areas are clustered according to similarity of their connectivity patterns in a parametric, continuous manner. Dimensionality reduction of whole-brain connections can provide a fingerprint of the connectome at the individual patient level (69), thereby representing a more realistic picture of stroke involving multiple functional domains. Using this approach, we recently showed that the location of a stroke lesion in whole-brain connectivity space is related to the degree of reorganization that occurs within the first week of stroke onset, as measured by whole-brain functional connectivity (70). This preliminary result supports the value of developing whole-brain connectivity models to characterize the widespread effects of localized lesions in detail.

Given the promising results of predicting NIBS response using electroencephalogram (EEG) connectivity (71) and the added value of functional connectivity changes to prognostic models of stroke outcome (72), we suggest that connectivity patterns may be useful biomarkers for response to NIBS. Going forward, the link between a connectome fingerprint and spontaneous recovery in multiple functional domains has to be established, followed by the predictive role of the connectome fingerprint prior to stimulation on the clinical response to NIBS, with the eventual goal of using this information to design NIBS protocols.

## Ongoing Neuronal Oscillations

Factors influencing response to NIBS can be subclassified into momentary (“state”) and phenotypic (“trait”) factors. Both can be assessed using properties of neuronal oscillations that reflect the cortex's susceptibility to stimulation.

An individual's response to a stimulation protocol is hard to predict. The exact same NIBS protocol may lead to excitatory, inhibitory, or no effects on motor evoked potentials in different individuals, even in the absence of pathology (14, 15, 73, 74). One way to reduce this variability is to align the stimulation with states in which the brain is most susceptible (“excitability states”) (75). There is evidence for the relevance of these states, including the observation that the variability of pre-stimulus alpha oscillations correlate with the variability of responses to TMS (76), power of sensorimotor mu (8–12 Hz oscillations above central-parietal electrodes) correlates with amplitude of motor evoked potentials (77), and synchronicity of mu oscillations in bilateral M1 is associated with stronger interhemispheric inhibition (75). These approaches are currently being pursued for targeted “state-dependent” NIBS (78, 79).

Properties of neuronal oscillations define instantaneous cortical reactivity to NIBS but are also subject-specific and highly heritable. This particularly relates to the power in the alpha band (80), and the temporal dynamics of the oscillations in alpha and beta bands (81). These results support the idea that beyond momentary states, properties of neuronal oscillations during rest can also represent a phenotypic trait.

The response to NIBS itself is also highly heritable (82), and intra-subject reliability of NIBS response is relatively high in healthy individuals (15). A recent EEG study showed that the temporal dynamics in the alpha band obtained before stimulation correlates on an individual level with the response to paired-pulse TMS in healthy individuals (83). These studies provide evidence that cortical plasticity is in part genetically determined, indicating a trait-like capacity of the brain to be modulated.

Studies show that neural networks might operate at the critical state, representing a balance between excitation and inhibition which is optimal for information processing (84–86). Critical states are also associated with the presence of long-range temporal correlations (LRTC) in the amplitude dynamics of neuronal oscillations (87). Given that LRTCs relate to cortical excitability (83), they are likely to be perturbed after stroke, as they are in several other neurological and psychiatric disorders (88–90). The patterns of perturbation may be linked to spontaneous recovery through reaching a compensatory state that effectively balances out the state of the network.

Trait-like properties of neuronal oscillations can be quantified using clinically accessible methods such as resting EEG. In our opinion, these may serve as potentially meaningful biomarkers for response to NIBS by accounting for variability in the cortex's susceptibility to stimulation in individual patients.

## New NIBS Approaches

Recent developments in NIBS technology will likely contribute to individualized therapy. Moving beyond single-area stimulation, targeting specific muscle groups that play different roles in post-stroke motor recovery (for example, finger flexors vs. extensors) will be possible using multi-locus TMS (91). This approach enables stimulation of multiple regions with high temporal precision, as it does not involve repositioning of the coil. The exact changes induced by NIBS on a sub-regional level (for example, in specific parts of the motor homunculus) can be predicted using advanced induced electrical field modeling (92, 93), further refining such targeting. Finally, deep brain structures, inaccessible using TMS and tDCS yet relevant for dexterity deficits and pathological synergies in stroke (94, 95), might be targeted using new non-invasive stimulation approaches such as transcranial focused ultrasound (96) or temporal interference (97). These technological advances along with the development and validation of meaningful biomarkers associated with response to NIBS can help advance the translation of NIBS while embracing the inevitable heterogeneity associated with stroke pathology.

## Author Contributions

SO-C, AK, MN, and VN project planning and conceptualization. SO-C, AK, and MN literature search and manuscript writing and revising. SO-C principle writing and revising. VN, BS, and AV manuscript revising or drafting.

### Conflict of Interest Statement

The authors declare that the research was conducted in the absence of any commercial or financial relationships that could be construed as a potential conflict of interest.
